# Daily standing time, dietary fiber, and intake of unsaturated fatty acids are beneficially associated with hepatic insulin sensitivity in adults with metabolic syndrome

**DOI:** 10.3389/fendo.2024.1272886

**Published:** 2024-06-26

**Authors:** Saara Laine, Tanja Sjöros, Taru Garthwaite, Miikka-Juhani Honka, Eliisa Löyttyniemi, Olli Eskola, Maria Saarenhovi, Petri Kallio, Mikko Koivumäki, Henri Vähä-Ypyä, Harri Sievänen, Tommi Vasankari, Jussi Hirvonen, Kirsi Laitinen, Noora Houttu, Kari Kalliokoski, Virva Saunavaara, Juhani Knuuti, Ilkka H.A. Heinonen

**Affiliations:** ^1^ Turku PET Centre, University of Turku, Åbo Akademi University and Turku University Hospital, Turku, Finland; ^2^ Department of Biostatistics, University of Turku, Turku, Finland; ^3^ Department of Clinical Physiology and Nuclear Medicine, University of Turku and Turku University Hospital, Turku, Finland; ^4^ Paavo Nurmi Center, University of Turku, Turku, Finland; ^5^ The UKK Institute for Health Promotion Research, Tampere, Finland; ^6^ Faculty of Medicine and Health Technology, Tampere University, Tampere, Finland; ^7^ Department of Radiology, University of Turku and Turku University Hospital, Turku, Finland; ^8^ Institute of Biomedicine, University of Turku, Turku, Finland; ^9^ Department of Medical Physics, Division of Medical Imaging, Turku University Hospital, Turku, Finland

**Keywords:** hepatic insulin sensitivity, hepatic glucose uptake, endogenous glucose production, sedentary behavior, physical activity

## Abstract

**Background:**

Obesity is associated with impaired glucose metabolism and hepatic insulin resistance. The aim was to investigate the associations of hepatic glucose uptake (HGU) and endogenous glucose production (EGP) to sedentary behavior (SB), physical activity (PA), cardiorespiratory fitness, dietary factors, and metabolic risk markers.

**Methods:**

Forty-four adults with metabolic syndrome (mean age 58 [SD 7] years, BMI ranging from 25–40kg/; 25 females) were included. HGU was measured by positron emission tomography during the hyperinsulinemic-euglycemic clamp. EGP was calculated by subtracting the glucose infusion rate during clamp from the glucose rate of disappearance. SB and PA were measured with hip-worn accelerometers (26 [SD3] days). Fitness was assessed by maximal bicycle ergometry with respiratory gas measurements and dietary intake of nutrients by 4-day food diaries.

**Results:**

HGU was not associated with fitness or any of the SB or PA measures. When adjusted for sex, age, and body fat-%, HGU was associated with whole-body insulin sensitivity (β=0.58), water-insoluble dietary fiber (β=0.29), energy percent (E%) of carbohydrates (β=-0.32), saccharose (β=-0.32), mono- and polyunsaturated fatty acids (β=0.35, β=0.41, respectively). EGP was associated with whole-body insulin sensitivity (β=-0.53), and low-density lipoprotein cholesterol [β=-0.31], and when further adjusted for accelerometry wear time, EGP was associated with standing [β=-0.43]. (p-value for all< 0.05).

**Conclusions:**

Standing more, consuming a diet rich in fiber and unsaturated fatty acids, and a lower intake of carbohydrates, especially sugar, associate beneficially with hepatic insulin sensitivity. Habitual SB, PA, or fitness may not be the primary modulators of HGU and EGP. However, these associations need to be confirmed with intervention studies.

## 1 Introduction

The liver is an important organ for normal whole-body glucose homeostasis by maintaining the balance between the uptake and storage of glucose and glucose production. Obesity is associated with impaired glucose metabolism (1), and hepatic insulin resistance (2), which are major indicators of developing metabolic syndrome (MetS) and non-alcoholic fatty liver disease (NAFLD) (1–3). It has been suggested that the liver is more prone to developing insulin resistance compared to muscle or adipose tissue and that the insulin resistance of muscle and adipose tissue may result from hepatic insulin resistance (4).

In healthy humans, hepatic glucose uptake (HGU) during insulin stimulation increase, and defective stimulation of HGU by insulin predisposes to postprandial hyperglycemia due to defective liver glycogen storage (5). Insulin-stimulated glucokinase activity is a key factor in liver glycogen storage (6). Glucokinase is an enzyme in hepatocytes that regulates carbohydrate metabolism by balancing rising and falling glucose levels, e.g., after meals or during fasting. During insulin stimulation, HGU mainly represents the activity of glucokinase, because the capacity of glucose transporters (GLUT-2) in the liver is large, and insulin either decreases or has no effect on GLUT-2 activation (7, 8).

Endogenous glucose production (EGP) is a net result of the release of glucose from glycogenolysis and gluconeogenesis that mainly happens in the liver (9). Thus, EGP assists in blood glucose level regulation and inhibits hypoglycemia by producing glucose that tissues require during fasting or physical exercise (9, 10). After a meal, insulin is secreted in a dose-dependent manner as a response to absorbed glucose, and EGP is suppressed (11). However, when glucose tolerance is impaired because of insulin resistance, hepatic tissue is not responding to insulin, glucose uptake is reduced, and EGP rates are increased (12). Thus, decreased HGU and increased EGP during insulin stimulation are specific markers of hepatic insulin resistance.

Both sedentary behavior (SB) and lack of physical activity (PA) are associated with obesity (13–15) and NAFLD (16). On the other hand, PA is known to have beneficial effects on insulin sensitivity in both healthy and insulin-resistant individuals (17). Understanding how habitual SB and PA are associated with hepatic insulin resistance would be important because it reflects an increased risk of developing metabolic diseases such as type 2 diabetes or NAFLD. Consequently, our primary aim was to examine the associations of HGU and, EGP to device-measured habitual SB and PA. Additionally, we examined the associations of HGU, and EGP to fitness, daily nutrient, and energy intake, body composition, liver fat content, and common markers of cardiometabolic risk.

## 2 Methods

### 2.1 Study design

This study used the baseline data of an intervention trial (Medical and physiological benefits of reduced sitting, ClinicalTrials.gov ID NCT03101228) performed at the Turku PET Centre, Turku, Finland, between April 2017, and August 2019. All participants gave written informed consent before enrollment in the study. The study was conducted according to good clinical practice and the Declaration of Helsinki and was approved by the Ethics Committee of the Hospital District of Southwest Finland (16/1810/2017).

### 2.2 Participants

The participants were sedentary middle-aged adults with MetS, BMI ranging from 25–40kg/m², who were recruited through bulletin boards and newspaper advertisements. Fulfillment of the metabolic syndrome criteria included three of the following symptoms (18): central obesity (waist circumference ≥ 94 cm for males and ≥ 80 cm for females), blood triglycerides ≥ 1.7 mmol/l, HDL-C cholesterol< 1.0 mmol/l for males and< 1.3 mmol/l for females, systolic blood pressure ≥130 and/or diastolic blood pressure ≥85 mmHg and fasting glucose > 5.6 mmol/l. The inclusion and exclusion criteria for participation are described in **Table 1**.

**Table 1:** Inclusion and exclusion criteria.

Inclusion criteria	Exclusion criteria
1) Age 40–65 years	1) History of a cardiac event
2) BMI 25–40 kg/m^2^	2) Insulin- or medically treated diabetes
3) Physically inactive (less than 120 minutes of moderate-intensity exercise per week reported during phone screening and initial physical activity questionnaires)	3) Any chronic disease or condition that could create a hazard to the subject’s safety, endanger the study procedures or interfere with the interpretation of study results
4) Sitting time ≥ 10 h/day or 60% of accelerometer wear time (measured by the accelerometer during screening)	4) Abundant use of alcohol (according to national guidelines)
5) Blood pressure< 160/100 mmHg	5) Use of narcotics, smoking of tobacco, or consuming snuff tobacco
6) Fasting plasma glucose< 7.0 mmol/l	6) Previous positron emission tomography (PET) or considerable exposure to radiation
7) Fulfillment of the metabolic syndrome criteria (18), including three of the following symptoms: - Central obesity (waist circumference ≥ 94 cm for males and ≥ 80 cm for females) - Blood triglycerides ≥ 1.7 mmol/l - HDL-C cholesterol< 1.0 mmol/l for males and< 1.3 mmol/l for females - Systolic blood pressure ≥130 and/or diastolic blood pressure ≥85 mmHg - Fasting glucose > 5.6 mmol/l	7) Diagnosed depressive or bipolar disorder 8) Inability to understand written Finnish

### 2.3 Measurements

#### 2.3.1 Whole-body insulin sensitivity

Whole-body insulin-stimulated glucose uptake (M-value) was assessed with the gold standard hyperinsulinemic-euglycemic clamp method after an overnight fast, as previously reported (19).

The hyperinsulinaemic-euglycaemic clamp was performed after at least 10 hours of fasting. A primed-constant insulin (Actrapid, 100 U/ml, Novo Nordisk, Bagsvaerd, Denmark) infusion rate was 160 mU/min/m2 of the participant’s body surface area during the first 4 min. From 4 to 7 min, the infusion rate was reduced to 80 mU/m2/min, and from 7 min to the end of the clamp, it was kept constant at 40 mU/m2/min. An exogenous 20% glucose infusion was started 4 min after the initiation of the insulin infusion, with a rate of ml/h per participant’s body mass (kg) x 0.5, e.g. for a person weighing 80 kg, the rate was 40 ml/h ≈ 8 g of glucose per hour. At 10 min, the glucose infusion was doubled, and after that further adjusted according to blood glucose concentration to keep it as close as possible to the level of 5 mmol/L. Arterialized venous blood samples were collected every 5 min during the first 30 min and at steady state every 10 min to determine the glucose concentration for adjusting the glucose infusion rate. The whole-body insulin-stimulated GU rate was calculated from the measured steady-state glucose values and glucose infusion rate starting from 20 min after the start of the hyperinsulinaemic-euglycaemic clamp. The outcome, M-value, represents whole-body glucose uptake as mg/kg/min.

#### 2.3.2 Hepatic glucose uptake

**Figure 1:**
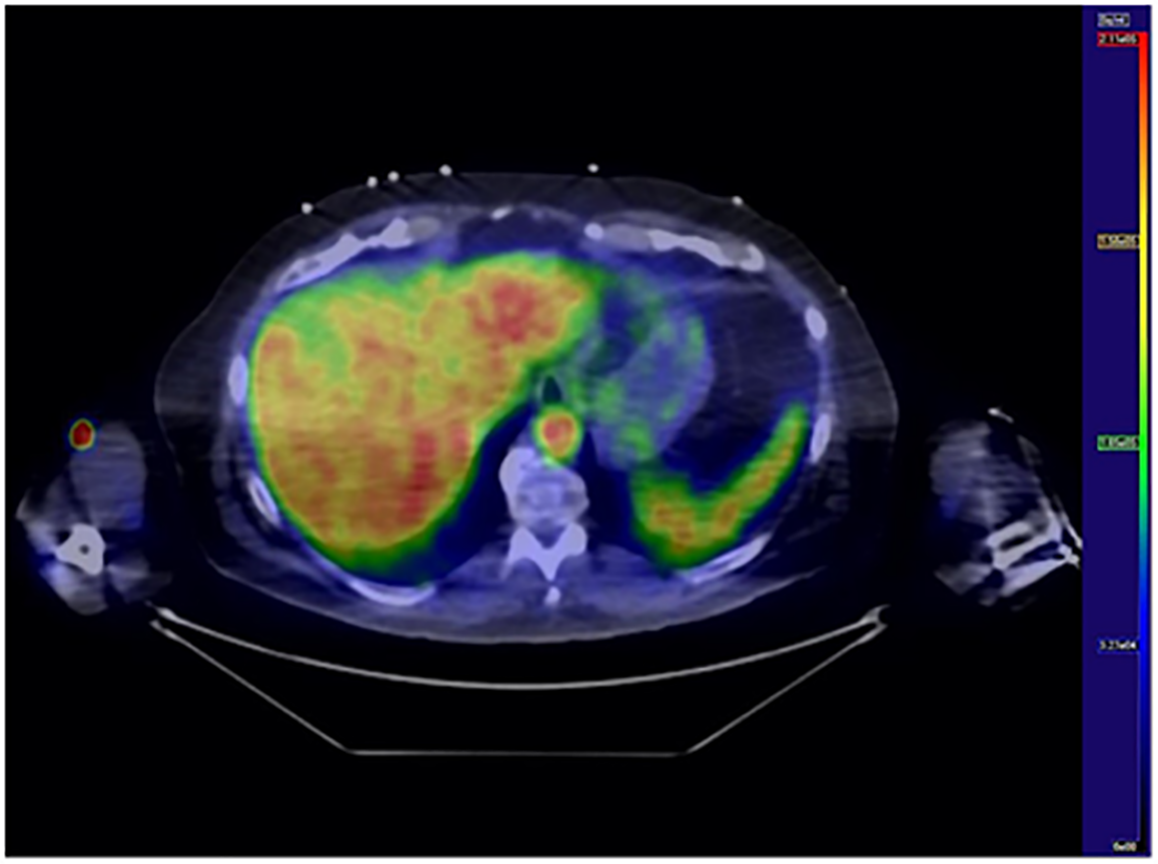
Hepatic glucose uptake. Transaxial [^18^F]FDG PET/CT image of the liver. Blue color represents low [^18^F]FDG uptake whereas red color represents high uptake.

HGU was measured during hyperinsulinemic-euglycemic clamp combined with 2-deoxy-2-[^18^F] fluoro-D-glucose ([^18^F]-FDG)-positron emission tomography (PET) imaging (**Figure 1**) using PET/computed tomography (CT) scanner (GE D690, GE Healthcare, Milwaukee, US). Radiotracer [^18^F]-FDG (20) was produced and [^18^F]-FDG uptake was analyzed as previously described (21). Hepatic region imaging started simultaneously with the tracer (168 [SD 11] MBq) injection into the antecubital vein 75 (SD 12) min after starting the clamp. The cumulative availability of the tracer in plasma (input function) was determined from the radioactivity in the left ventricle of the heart during the first 40 min of PET imaging and from blood samples collected at approximately 50 and 70 min after the injection. All data were corrected for dead-time, decay, and measured photon attenuation. Dynamic PET scan was reconstructed with an iterative reconstruction method.

^18^F-FDG activity in the hepatic tissue was measured by drawing a region of interest (ROI) in the right lobe of the liver using a CT image as an anatomic reference. The PET/CT images were analyzed with Carimas (v.2.71, Turku PET Centre, Turku, Finland). HGU (µmol/ml/min) was calculated by multiplying the tissue fractional phosphorylation rate (Ki) by the average plasma glucose concentration during scanning. Lumped constant (LC) for the liver is 1.0. The software Carimas (v.2.71, Turku PET Centre, Turku, Finland) was used to analyze PET/CT images of the liver. Plasma radioactivity was measured with an automatic gamma counter (Wizard 1480 3”, Wallac, Turku, Finland).

#### 2.3.3 Endogenous glucose production

EGP was calculated by subtracting the exogenous glucose infusion rate (GIR) corrected by space correction (22) from the glucose rate of disappearance (Rd) during the hyperinsulinemic-euglycemic clamp (23), according to the following equation;


$$\text{EGP}=\text{Rd}+{\text{V}}_{\text{glucose}}\;\text{x}\frac{\Delta glucose}{\Delta T}-GIR$$

*Rd* = rate of disappearance (µmol/kg/min)
*V_glucose_* = estimated glucose distribution volume (0.19 l/kg)
*Δglucose* = change in glucose from [^18^F]-FDG injection to the end of blood sampling (mmol/l)
*ΔT* = time from [^18^F]-FDG injection to the end of blood sampling (min)
*GIR* = glucose infusion rate (µmol/kg/min)

Rd was calculated using [^18^F]-FDG clearance corrected by tracer lost to urine (24);


$$\text{Rd}=\frac{dose_{FDG}-urine_{FDG}}{AUC_{FDG}}\;\text{x}\;glucose_{avg}$$

Dose_FDG_ = radioactivity of the injected [^18^F]-FDG
Urine_FDG_ = secreted [^18^F]-FDG to urine from the tracer injection until voiding bladder at the end of the study
AUC_FDG_ = area under the curve representing [^18^F]-FDG from the tracer injection to infinity
Glucose_avg_ = average glycemia during the interval between the time of [^18^F]-FDG injection and the end of blood sampling.

#### 2.3.4 Sedentary behavior and physical activity

SB and PA were measured for four weeks with validated hip-worn tri-axial accelerometers (UKK AM30, UKK-Institute, Tampere, Finland) and analysis methods, as previously reported (25). Briefly, accelerometers were worn during waking hours for four consecutive weeks (except during water-based activities). The collected accelerometer data was analyzed in six-second epochs and SB (sitting and lying together), standing, light physical activity (LPA), moderate-to-vigorous physical activity (MVPA), steps, and breaks in SB were defined using mean amplitude deviation (MAD) (26) and angle for posture estimation (APE) methods (27). The daily means for the following SB and PA variables were calculated: SB (h/day), breaks in SB (times/day), standing (h/day), daily steps (number/day), LPA (h/day), MVPA (h/day), and total PA (LPA and MVPA together, h/day). For valid data collection, a daily wear time of 10–19 h and at least 4 days of valid measurements were required. Daily measurement time exceeding 19 h indicates that the participant has likely slept with the accelerometer and measurement hours exceeding 19 h per day, were subtracted from the SB time.

#### 2.3.5 Cardiorespiratory fitness

Maximal oxygen consumption (VO_2max_) measurements were conducted after the participants had passed a thorough physical examination and electrocardiographical measurements. VO_2max_ was determined by bicycle ergometry (eBike EL Ergometer + CASE v6.7, GE Medical Systems Information Technologies, Inc. Milwaukee, WI, USA) with direct respiratory gas measurements (Vyntus CPX, CareFusion, Yorba Linda, CA, USA). VO_2max_ per fat-free mass (FFM) (ml/min/kg_FFM_), and maximal load (Wmax) were also determined. The exercise workload was started at 25 W and increased by 25 W every three minutes until exhaustion. Participants were instructed to maintain a pace of 60–65 rpm throughout the test. Blood pressure and perceived exertion on the Borg scale (28) were measured one minute after each increase in workload. Maximal oxygen consumption (VO_2max_) was determined if one criterion was met: respiratory exchange ratio > 1.0, a plateau in VO2, or heart rate within ±10 bpm of the age-predicted maximum. VO_2max_ was defined as the highest one-minute average in ml/min/kg.

#### 2.3.6 Dietary intake

Daily total energy intake and intakes of carbohydrates (CHO), protein, fat, alcohol, saturated fatty acids (SFA), monounsaturated fatty acids (MUFA), polyunsaturated fatty acids (PUFA), saccharose, fiber, and water-insoluble dietary fiber (WIDF) were calculated from 4-day food diaries (including one weekend day), and analyzed by a nutritionist with computerized software (AivoDiet 2.2.0.1, Aivo, Turku) that utilized Finnish Food Composition Database Fineli (29).

#### 2.3.7 Body composition, anthropometry, blood pressure

Validated (30) air displacement plethysmography (the Bod Pod system, COSMED, Inc., Concord, CA, USA) with predicted thoracic gas volume was used to estimate body composition (body fat-%) after fasting for at least four hours. Participants were advised not to exercise or take a shower beforehand on the day of the measurement. After emptying the bladder, participants entered the measurement chamber wearing a tight cap and underwear or a swimming suit. Body weight was measured by a scale (Seca 797, Vogel & Halke, Hamburg, Germany) in light clothing and body height barefooted with a wall-mounted stadiometer. Body mass index (BMI) was calculated from the measured weight and height in kg/m^2^. Waist circumference (WC) was measured with a flexible measuring tape midline between the iliac crest and the lowest rib, and the measurement was repeated twice or until the same measure was obtained twice. Blood pressure and resting heart rate were measured using a digital blood pressure monitor (Apteq AE701f, Rossmax International LtD, Taipei, Taiwan) in a seated position after at least 5 min of sitting. The mean of 2–3 measurements was used as the outcome measure. The anthropometric variables were measured under standard conditions. All the measurements were performed by the same researcher to ensure standardized measurements.

#### 2.3.8 Liver fat content

Liver fat content (LFC) was measured by magnetic resonance spectroscopy (MRS) and magnetic resonance imaging (MRI), based on two-point Dixon [2PD] method using a Philips 3 Tesla system (Ingenuity TF PET/MR) with a Q-Body coil. Because of the MRI scanner replacement during this study, MRS and MRI quantification of LFC of seven participants were conducted with Siemens Magnetom Skyra fit 3 T MRI system (Siemens Healthcare, Erlangen, Germany) with Siemens Body 30 and 18 channel coils, and 32 channel Spine coil.

The spectra were acquired using stimulated echo acquisition mode (STEAM) 1H MRS with parameters: repetition time (TR)/echo time (TE)/mixing time (TM) = 2000/11/17 ms, 4 averages, 2048 samples, spectral bandwidth 2000 Hz, and acquisition volume 20 x 20 x 30 mm3. Data were acquired during 12 breath holds. Water saturation was done with chemical shift selective (CHESS) with 50 Hz bandwidth. The duration of the scan was 3:12.0. The 3D T1-fast field echo sequence was acquired in the axial plane with parameters: TR/TE1/TE2 = 2.8/0.81/1.8 ms, flip angle 10°, FOV 510 mm x 510 mm, imaging matrix 188 x 188. Data was reconstructed to voxel size 2.13 x 2.13 x 4 mm3. Respiratory gating was used in the thorax–upper abdomen area.

MRS and MRI quantification of LFC conducted with Siemens Magnetom Skyra fit 3 T MRI system (Siemens Healthcare, Erlangen, Germany) with Siemens Body 30 and 18 channel coils, and 32 channel Spine coil. The spectra were acquired with point resolved spectroscopy (PRESS) 1H MRS with parameters TR/TE = 4000/30 ms, averages 32, 1024 samples, acquisition volume 20 x 20 x 20 mm3. Respiratory motion was controlled using a navigator. Water saturation was done with a 35 Hz bandwidth. The duration of the scan was 3:10. The 3D gradient echo volumetric interpolated breath-hold examination (VIBE Dixon) sequence was acquired in the axial plane with parameters: TR/TE1/TE2 = 3.97/1.23/2.46, flip angle 9°, and voxel size 2 x 2 x 2 mm3. Breath holds were used in the thorax–upper abdomen area. Controlled aliasing in parallel imaging results in higher acceleration (CAIPIRINHA) was used. Water signal and fat signal images were used to calculate the fat fraction map (31) from which the LFC was determined; an MRI image was used as an anatomical reference. LC Model (Version 6.3–0C) was used to quantify liver fat with ‘liver-4’ as a spectrum type. Lipid signals 1.6 ppm, 1.3 ppm, and 0.9 were used. The fat and water signals were corrected due to the difference in T2 decay (32, 33) and molar concentrations of 1H nuclei in fat and water as reported before (34, 35). Liver fat content was defined as fat in relation to the total weight of liver tissue (32).

MRI images were analyzed using Carimas software version 2.10 (http://turkupetcentre.fi/). Four representative three-dimensional regions of interest (ROIs) were drawn manually on the sections of the liver (left lateral and medial section, right anterior and posterior section) avoiding the main portal veins. The results were volume corrected with the following formula: mean volume of one section x (total volume (mm3) of one section/total volume of all sections).

#### 2.3.9 Blood sampling

Venous blood samples were drawn after at least 10 h of fasting. Plasma glucose was determined by enzymatic reference method with hexokinase GLUC3 and plasma insulin was determined by electrochemiluminescence immunoassay (Cobas 8000 e801, Roche Diagnostics GmbH, Mannheim, Germany). Hemoglobin A_1c_ (HbA_1c_) was determined by turbidimetric inhibition immunoassay (Cobas 6000 c501, Roche Diagnostics GmbH, Mannheim, Germany). Plasma triglycerides, total cholesterol, low-density lipoprotein cholesterol (LDL-C), and high-density lipoprotein cholesterol (HDL-C) by enzymatic colorimetric tests (Cobas 8000 c702, Roche Diagnostics GmbH, Mannheim, Germany). Alanine aminotransferase (ALT) and aspartate aminotransferase (AST) were determined by the photometric IFCC (International Federation of Clinical Chemistry) method (Cobas 8000 c702 and c 502 Analyzer, Roche Diagnostics GmbH, Mannheim, Germany), and γ-glutamyltransferase (GGT) by enzymatic colorimetric tests and assay (Cobas 8000 c702, Roche Diagnostics GmbH, Mannheim, Germany). All the samples were analyzed at the Turku University Hospital Laboratory. Homeostatic model assessment of insulin resistance (HOMA-IR) was calculated using the formula: fasting glucose (mmol/l) x fasting insulin (mU/l)/22.5 (36).

#### 2.3.10 Statistical methods

The associations of HGU and EGP (dependent variables) with SB and PA measures, fitness, nutrient intake, and cardiometabolic health markers (independent variables) were examined with linear mixed models. The first model was adjusted for sex and age (model 1) and the second model additionally for body fat-% (model 2). Additionally, all the models with SB and PA outcomes were adjusted for accelerometer wear time. An unpaired t-test was used to compare the sexes and EGP according to standing time (≤1h 45min [n=22] vs. >1h 45 min [n=21], and daily sedentary time (≤ 10.0 h/day [n=21] vs. >10.0 h/day [n=22]), and HGU according to daily fiber consumption (≤ 18 g/day [n=21] vs. >18 g/day [n=22]) and water-insoluble dietary fiber consumption (≤ 13 g/day [n=21] vs. >13 g/day [n=22]). The normal distribution of the residuals was assessed by visual evaluation and the Shapiro-Wilk test, and logarithmic transformations were used when necessary to fulfill the normal distribution assumption. Multicollinearity was controlled for with variance inflation factors, which all were below five indicating no multicollinearity issues. Missing data was handled by pairwise deletion. A power calculation to determine the sample size was performed for the primary outcome (whole-body insulin sensitivity) of the sedentary behavior reduction intervention study (NCT03101228), from which the baseline imaging measurements form the data of this study. HGU and EGP measures of one participant were missing due to technical difficulties. VO_2max_ measures of two participants were excluded because the test was stopped before reaching volitional exhaustion (due to knee pain or difficulties in breathing), and the results of one participant were lost due to technical problems. MRS-measured LFCs of three participants were missing due to image artifacts and MRS and MRI-measured LFCs of one participant were missing due to technical challenges with the scanner. Data are expressed as mean and standard deviation (SD), standardized β coefficients, and 95% confidence interval (CI) values. The level of statistical significance was set at 5% (two-tailed). All analyses were conducted with the JMP^®^, pro 13.1 for Windows (SAS Institute Inc., Cary, NC, USA), and with GraphPad Prism 5.01 (GraphPad Software, San Diego, CA). The figures were created with GraphPad Prism 5.01 (GraphPad Software, San Diego, CA) and JMP^®^, pro 16.0 (SAS Institute Inc., Cary, NC, 1989–2023).

## 3 Results

### 3.1 Characteristics of the participants

In total, 263 individuals volunteered, of which 151 participated in the screening measurements and 44 participants were included in the study. Participants’ baseline characteristics grouped by sex are presented in **Table 2**. Sixty-six percent of the participants were obese (BMI ≥ 30 kg/m^2^), and 34% were overweight (BMI 25.0 to< 30). Participants had medication for elevated blood pressure (n=24) and for elevated blood cholesterol (n=9). Some participants also reported use of hormonal replacement therapy medication (n=7), pain medication (n=5), anticoagulants (n=5), thyroid medication (n=4), gastrointestinal medication (n=4), allergy or asthma medication (n=4), antidepressants (n=3), sleep medication (n=3), medication for urinary problems (n=2), osteoarthritis medication (n=1) and medication for restless legs syndrome (n=1).

**Table 2:** Characteristics of the study participants by sex. If not otherwise stated, the results are reported as mean (SD).

	Males	Females
n, (% of total)	19 (43)	25 (57)
Age, years	58 (6.0)	57 (7.3)
Anthropometrics		
BMI, kg/m^2^	31.8 (4.7)	32.5 (4.1)
Waist circumference, cm	115.2 (13.2)	108.4 (9.5)
Body fat-%	37.5 (7.8)	48.1 (3.8) *
HGU and EGP		
HGU, µmol/100 ml/min	2.2 (1.0)	3.0 (1.4)
EGP, µmol/kg/min	-0.2 (8.4)	-1.9 (7.7)
Liver fat content		
MRS-measured LFC, %#	5.1 (3.8)	2.6 (3.0) *
MRI-measured LFC, %#	11.9 (5.8)	8.0 (3.9) *
Health measurements		
Systolic blood pressure, mmHg	140 (14)	146 (13)
Diastolic blood pressure, mmHg	89 (9)	89 (6)
Blood pressure medication, n (%)	13 (68)	9 (36)
Cholesterol medication, n (%)	4 (21)	4 (16)
Resting heart rate, bpm	67 (6)	68 (10)
f-Glucose, mmol/l	5.9 (0.5)	5.7 (0.2)
f-Insulin, µmol/l	16.2 (9.4)	10.4 (4.3) *
HbA_1c_, mmol/mol	37.8 (2.5)	36.8 (2.7)
HOMA-IR	4.3 (2.7)	2.7 (1.1) *
M-value, mg/kg/min	2.9 (2.8)	3.6 (2.1)
Triglycerides, mmol/l	1.4 (0.5)	1.3 (0.7)
Cholesterol, mmol/l	4.4 (0.7)	4.9 (1.1)
HDL-C, mmol/l	1.1 (0.3)	1.4 (0.3) **
LDL-C, mmol/l	2.9 (0.7)	3.2 (1.0)
ALT, U/l	36 (18)	27 (12)
AST, U/l	31 (12)	23 (5) *
GGT, U/l	34 (21)	25 (17)
Accelerometry		
Sedentary time, h/day	10.3 (0.9)	10.1 (0.9)
Accelerometry, days	26 (2)	27 (3)
Wear time, h/day	14.3 (1.0)	14.9 (0.8) *
Breaks in sedentary, time/day	24 (5)	32 (8) **
Standing, h/day	1.5 (0.4)	2.0 (0.5) ***
Daily steps	5194 (2134)	4986 (1382)
LPA, h/day	1.6 (0.5)	1.9 (0.3) *
MVPA, h/day	1.0 (0.4)	0.9 (0.2)
PA, h/day	2.6 (0.7)	2.8 (0.4)
Cardiorespiratory fitness		
VO_2max_, ml/min/kg	24.7 (5.6)	21.3 (3.4) *
VO_2max_, ml/min/kg_FFM_	39.5 (7.4)	41.0 (5.6)
Maximal load, W	145.7 (36.6)	120.1 (28.4) *
Nutrition		
Total EI, kcal/day	1884.0 (377.8)	1742.5 (341.9)
Protein, % of daily EI	17.9 (2.9)	17.8 (2.8)
Carbohydrates, % of daily EI	39.7 (7.8)	40.7 (6.0)
Fat, % of daily EI	38.8 (6.2)	38.0 (5.2)
Alcohol, % of daily EI	1.7 (3.5)	1.4 (1.6)
SFA, % of daily EI	14.5 (3.3)	13.7 (2.4)
MUFA, % of daily EI	13.5 (3.7)	12.9 (2.0)
PUFA, % of daily EI	5.6 (1.5)	5.9 (1.2)
Saccharose, % of daily EI	7.3 (3.2)	8.3 (4.2)

Significant p-values; * p< 0.05, ** p< 0.01, *** p< 0.001. Sex difference in a t-test (or Fisher’s exact test, when applicable).

Results published previously by Laine et al. (37).

BMI, body mass index; HGU, hepatic glucose uptake; EGP, endogenous glucose uptake; MRS, magnetic resonance spectroscopy; LFC, liver fat content; MRI, magnetic resonance imaging; HbA_1c_, hemoglobin A1c; HOMA-IR, homeostatic model assessment for insulin resistance; M-value, whole-body insulin sensitivity; HDL-C, high-density lipoprotein cholesterol; LDL-C, low-density lipoprotein cholesterol; ALT, alanine aminotransferase; AST, aspartate aminotransferase; GGT, γ-glutamyltransferase; LPA, light physical activity; MVPA, moderate to vigorous physical activity; PA, physical activity (LPA + MVPA); VO_2max_, maximal oxygen consumption; FFM, fat-free mass; EI, energy intake; SFA, saturated fatty acids; MUFA, monounsaturated fatty acids; PUFA, polyunsaturated fatty acids.

Bold values represent significance level < 0.05.

### 3.2 Associations of HGU and EGP with SB, PA, and fitness

**Figure 2:**
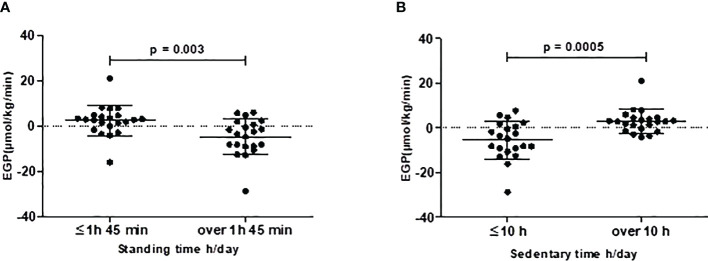
Endogenous glucose production (EGP) is better with **(A)** standing time >1h 45 min (n=21) vs ≤1h 45min (n=22) and with **(B)** sedentary time >10.0 h/day (n=22) vs. ≤10.0 h/day (n = 21). Black dots represent individual participants, and black lines with error bars indicate means (SD).

When adjusted for age, sex, and accelerometer wear time, HGU was not associated with any of the SB, PA, or fitness variables (model 1, **Table 3**). Associations remained non-significant when body fat-% was added to the model (model 2, **Table 3**). In the age-, sex- and accelerometer wear time-adjusted model, EGP was negatively associated with standing time (h/day), and positively with SB time (h/day) (model 1, **Table 3**). When further adjusted for body fat-%, the association between EGP and standing time remained significant; however, the association between EGP and SB time turned non-significant (model 2, **Table 3**). Additionally, EGP was better with standing time >1h 45 min compared to ≤1h 45min [EGP -4.8 (−8.1, -1.6) vs. +2.4 (−0.8, 5.5), respectively, p = 0.003] (**Figure 2A**), and with sedentary time ≤10.0 h/day compared to >10.0 h/day [EGP -5.4 (-9.3, -1.6*)* vs. +3.0 (0.6, 5.3), respectively, p = 0.0005] (**Figure 2B**). When adjusted for age and sex, EGP associated negatively with VO_2 max_ (ml/min/kg) (model 1, **Table 3**). However, when body fat-% was included in the model, the association turned non-significant (model 2, **Table 3**).

**Table 3:** Age-, sex- accelerometry wear time and body fat-% -adjusted linear mixed regression estimates (standardized β coefficients [95% CI]) between HGU, EGP, sedentary behavior, physical activity, and cardiorespiratory fitness.

	HGU^a^ (µmol/100 ml/min)				EGP (µmol/kg/min)			
	Model 1		Model 2		Model 1		Model 2	
	β	p	β	p	β	p	β	p
Sedentary time, h/day	0.07 (-0.28, 0.42)	0.68	0.18 (-0.17, 0.54)	0.31	0.39 (0.02, 0.75)	**0.04**	0.26 (-0.10, 0.63)	0.154
Breaks in SB, times/day	0.16 (-0.19, 0.52)	0.36	0.1 (-0.25, 0.46)	0.56	-0.24 (-0.63, 0.14)	0.21	-0.15 (-0.52, 0.22)	0.41
Standing, h/day	-0.02 (-0.38, 0.34)	0.90	-0.11 (-0.47, 0.25)	0.54	-0.53 (-0.88, -0.18)	**0.004**	-0.43 (-0.78, -0.08)	**0.02**
Steps, number/day	0.14 (-0.18, 0.46)	0.39	0.02 (-0.32, 0.37)	0.89	-0.08 (-0.43, 0.27)	0.65	0.14 (-0.22, 0.5)	0.44
LPA, h/day	-0.15 (-0.49, 0.20)	0.39	-0.16 (-0.50, 0.17)	0.33	-0.16 (-0.53, 0.22)	0.41	-0.13 (-0.48, 0.22)	0.45
MVPA, h/day	0.03 (-0.29, 0.35)	0.86	-0.08 (-0.42, 0.25)	0.62	-0.05 (-0.4, 0.3)	0.77	0.13 (-0.22, 0.48)	0.45
PA, h/day	-0.09 (-0.42, 0.25)	0.60	-0.17 (-0.50, 0.16)	0.31	-0.14 (-0.5, 0.22)	0.44	-0.03 (-0.38, 0.32)	0.88
VO_2max_, ml/min/kg	0.13 (-0.24, 0.51)	0.48	-0.21 (-0.69, 0.28)	0.39	-0.42 (-0.8, -0.04)	**0.03**	-0.12 (-0.62, 0.38)	0.62
VO_2max_, ml/min/kgFFM	-0.19 (-0.55, 0.17)	0.29	-0.2 (-0.55, 0.14)	0.24	-0.02 (-0.41, 0.36)	0.90	-0.01 (-0.36, 0.35)	0.97
Maximal load, W	0.03 (-0.37, 0.43)	0.87	-0.13 (-0.54, 0.28)	0.52	-0.16 (-0.58, 0.25)	0.43	0.03 (-0.38, 0.45)	0.87

HGU, hepatic glucose uptake; EGP, endogenous glucose production; LPA, light physical activity; MVPA, moderate to vigorous physical activity; PA, physical activity (LPA and MVPA together); VO_2max_, maximal oxygen consumption; FFM, fat-free mass.

a = log10 transformed variables.

Model 1 adjusted for age, sex, and accelerometry wear time.

Model 2 adjusted for age, sex, accelerometry wear time, and body fat-%.

Bold values represent significance level < 0.05.

### 3.3 Associations of HGU and EGP with nutrient intake

**Figure 3:**
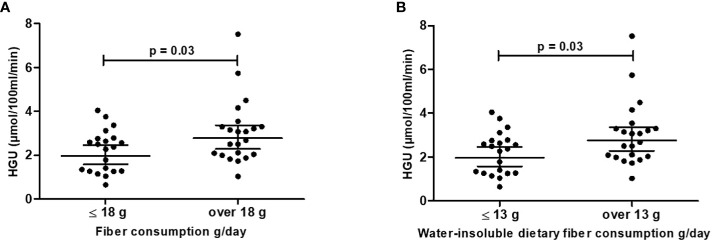
Hepatic glucose uptake (HGU) is better with **(A)** fiber consumption >18 g/day (n=22) vs. ≤ 18 g/day (n=21) and **(B)** water-insoluble dietary fiber consumption >13 g/day (n=22) vs. ≤ 13 g/day (n = 21). Black dots represent individual participants, and black lines with error bars indicate means (SD). HGU values are log10 transformed (means are back-transformed geometric model-based means [95% CI]).

In the age- and sex-adjusted model HGU was positively associated with daily total fiber intake (g/day) and with water-insoluble dietary fiber (WIDF, g/day) (model 1, **Table 4**). The association between HGU and WIDF remained significant when body fat-% was added to the model (model 2, **Table 4**). Also, HGU was better with fiber consumption > 18 g/day compared to ≤ 18g/day [HGU 3.0 (± 0.3) vs. 2.2 (± 0.2), respectively, p = 0.03] (**Figure 3A**), and with consumption of water-insoluble dietary fiber > 13 g/day compared to ≤ 13g/day [HGU 3.0 (± 0.3) vs. 2.2 (± 0.2), respectively, p = 0.03] (**Figure 3B**). Additionally, in the age-, sex- and body fat-%-adjusted model, HGU was negatively associated with intakes of carbohydrates (CHO, E%) and saccharose (E%) and positively associated with the proportion of mono- and polyunsaturated fatty acids (MUFA, E%; PUFA, E%; respectively) (model 2, **Table 4**).

**Table 4:** Age- sex- and body fat-% -adjusted linear mixed regression estimates (standardized β coefficients [95% CI]) between HGU, EGP, and dietary intake.

	HGU^a^ (µmol/100 ml/min)				EGP (µmol/kg/min)			
	Model 1		Model 2		Model 1		Model 2	
	β	p	β	p	β	p	β	p
Total EI, kcal/day	0.06 (-0.27, 0.38)	0.72	0.07 (-0.24, 0.37)	0.67	0.02 (-0.33, 0.36)	0.93	0.01 (-0.31, 0.32)	0.97
Protein, % of daily EI	0.03 (-0.28, 0.34)	0.84	0.09 (-0.21, 0.39)	0.54	0.02 (-0.31, 0.34)	0.92	-0.06 (-0.36, 0.24)	0.69
CHO, % of daily EI	-0.17 (-0.48, 0.14)	0.27	-0.32 (-0.62, -0.01)	**0.04**	-0.04 (-0.38, 0.29)	0.80	0.12 (-0.21, 0.44)	0.47
Fat, % of daily EI	0.16 (-0.16, 0.48)	0.31	0.3 (-0.01, 0.61)	0.06	0.02 (-0.32, 0.36)	0.92	-0.14 (-0.46, 0.19)	0.4
Alcohol, % of daily EI	-0.02 (-0.32, 0.29)	0.91	-0.01 (-0.30, 0.28)	0.94	0.07 (-0.25, 0.4)	0.65	0.06 (-0.23, 0.36)	0.67
SFA, % of daily EI	0.01 (-0.31, 0.34)	0.95	-0.03 (-0.34, 0.28)	0.86	0.04 (-0.30, 0.38)	0.82	0.09 (-0.22. 0.41)	0.56
MUFA, % of daily EI	0.17 (-0.14, 0.47)	0.27	0.35 (0.05, 0.65)	**0.02**	0.02 (-0.30, 0.35)	0.89	-0.17 (-0.5, 0.15)	0.29
PUFA, % of daily EI	0.24 (-0.06, 0.54)	0.11	0.41 (0.13, 0.70)	**0.01**	-0.06 (-0.38, 0.27)	0.72	-0.25 (-0.56, 0.07)	0.12
Saccharose, % of daily EI	-0.21 (-0.51, 0.09)	0.17	-0.32 (-0.60, -0.03)	**0.03**	-0.04 (-0.36, 0.28)	0.80	0.08 (-0.23, 0.39)	0.61
Fiber all, g/day	0.32 (0.03, -0.60)	**0.03**	0.26 (-0.03, 0.54)	0.08	-0.16 (0.03, -0.60)	0.31	-0.07 (-0.37, 0.24)	0.67
Water-insoluble dietary fiber, g/day	0.35 (0.07, 0.64)	**0.02**	0.29 (0.01, 0.58)	**0.046**	-0.13 (-0.45, 0.19)	0.42	-0.02 (-0.33, 0.29)	0.88

HGU, hepatic glucose uptake; EGP,endogenous glucose production; EI, energy intake; CHO, carbohydrates; SFA, saturated fatty acids; MUFA, monounsaturated fatty acids; PUFA, polyunsaturated fatty acids.

a = log10 transformed variables.

Model 1 adjusted for age and sex.

Model 2 adjusted for age, sex, and body fat-%.

Bold values represent significance level < 0.05.

In the sex-, and age-adjusted model, EGP was not associated with any of the dietary variables (model 1, **Table 4**), and the associations remained non-significant when body fat-% was added to the model (model 2, **Table 4**).

### 3.4 Associations of HGU and EGP with cardiometabolic health markers and liver fat content

When adjusted for age and sex, HGU was associated negatively with body fat-%, fasting insulin, HOMA-IR, and positively with M-value (model 1, **Table 5**). After further adjustment for body fat-%, only the association between HGU and M-value remained significant (model 2, **Table 5**). When adjusted for age and sex, EGP was associated positively with body fat-%, waist circumference, fasting insulin, HOMA-IR, and negatively with M-value and LDL-C (model 1, **Table 5**). After further adjustment for body fat-%, the associations between EGP and M-value and LDL-C remained significant (model 2, **Table 5**). Neither HGU nor EGP was associated with liver enzymes ALT, AST or GGT, or MRS- and MRI-measured liver fat content in any of the models (model 1–2, **Table 5**).

**Table 5:** Age-, sex- and body fat-% -adjusted linear mixed regression estimates (standardized β coefficients [95% CI]) between HGU, EGP, cardiometabolic health markers, and liver fat content.

	HGU^a^ (µmol/100 ml/min)				EGP (µmol/kg/min)			
	Model 1		Model 2		Model 1		Model 2	
	β	p	β	p	β	p	β	p
Body fat, %	-0.42 (-0.80, -0.04)	**0.03**			0.56 (0.17, 0.95)	**0.01**		
weight, kg	-0.15 (-0.47, 0.18)	0.37	0.06 (-0.32, 0.43)	0.76	0.20 (-0.14, 0.54)	0.23	-0.06 (-0.44, 0.32)	0.74
BMI, kg/m2	-0.22 (-0.52, 0.07)	0.14	-0.01 (-0.41, 0.39)	0.95	0.27 (-0.05, 0.58)	0.09	-0.04 (-0.45, 0.37)	0.84
Waist circumference, cm	-0.13 (-0.45, 0.19)	0.43	0.15 (-0.25, 0.55)	0.45	0.36 (0.03, 0.68)	**0.03**	0.12 (-0.29, 0.53)	0.56
Systolic blood pressure, mmHg	-0.1 (-0.42, 0.22)	0.54	-0.04 (-0.35, 0.27)	0.81	0.19 (-0.14, 0.52)	0.25	0.11 (-0.2, 0.43)	0.47
Diastolic blood pressure, mmHg	0.17 (-0.14, 0.47)	0.28	0.24 (-0.04, 0.53)	0.10	0.18 (-0.14, 0.50)	0.26	0.1 (-0.21, 0.40)	0.53
Resting heart rate, bpm	0.21 (-0.10, 0.52)	0.17	0.21 (-0.09, 0.51)	0.16	0.08 (-0.25, 0.41)	0.61	0.08 (-0.22, 0.39)	0.58
f-Glucose, mmol/l	-0.14 (-0.46, 0.17)	0.37	-0.09 (-0.4, 0.21)	0.54	0.32 (0.004, 0.64)	0.052	0.26 (-0.05, 0.56)	0.09
f-Insulin, mU/l	-0.34 (-0.65, -0.02)	**0.04**	-0.23 (-0.57, 0.11)	0.18	0.38 (0.04, 0.71)	**0.03**	0.22 (-0.13, 0.57)	0.21
HOMA-IR	-0.33 (-0.65, -0.01)	**0.04**	-0.23 (-0.57, 0.11)	0.18	0.38 (0.05, 0.72)	**0.02**	0.24 (-0.10, 0.59)	0.16
M-value	0.55 (0.30, 0.80)	**<.0001**	0.58 (0.25, 0.90)	**0.001**	-0.59 (-0.85, -0.32)	**<.0001**	-0.53 (-0.88, -0.19)	**0.003**
HbA1c, mmol/mol	0.04 (-0.28, 0.37)	0.79	0.13 (-0.19, 0.45)	0.41	0.04 (-0.31, 0.38)	0.84	-0.07 (-0.4, 0.25)	0.65
Triglycerides, mmol/l	-0.02 (-0.33, 0.28)	0.88	-0.01 (-0.30, 0.28)	0.95	0.02 (-0.30, 0.34)	0.89	0.004 (-0.29, 0.30)	0.98
Cholesterol, mmol/l	0.27 (-0.03, 0.58)	0.08	0.25 (-0.04, 0.54)	0.09	-0.29 (-0.61, 0.03)	0.08	-0.26 (-0.56, 0.04)	0.08
HDL-C, mmol/l	0.26 (-0.07, 0.59)	0.12	0.22 (-0.10, 0.54)	0.17	0.03 (-0.33, 0.40)	0.85	0.09 (-0.25, 0.42)	0.60
LDL-C, mmol/l	0.18 (-0.12, 0.48)	0.24	0.17 (-0.12, 0.46)	0.24	-0.32 (-0.63, -0.01)	**0.046**	-0.31 (-0.59, -0.02)	**0.04**
ALT, U/l	0.02 (-0.30, 0.34)	0.90	0.13 (-0.19, 0.45)	0.41	0.27 (-0.06, 0.59)	0.11	0.15 (-0.17, 0.47)	0.36
AST, U/l	0.21 (-0.13, 0.55)	0.21	0.27 (-0.06, 0.59)	0.10	0.01 (-0.36, 0.38)	0.96	-0.06 (-0.40, 0.28)	0.73
GGT, U/l	0.13 (-0.18, 0.44)	0.40	0.18 (-0.12, 0.47)	0.23	0.12 (-0.21, 0.44)	0.48	0.06 (-0.25, 0.36)	0.71
MRS-measured LFC, %	-0.06 (-0.40, 0.28)	0.74	-0.003 (-0.34, 0.33)	0.99	0.13 (-0.23, 0.50)	0.46	0.09 (-0.28, 0.46)	0.62
MRI-measured LFC, %	-0.38 (-0.39, 0.31)	0.83	0.18 (-0.20, 0.55)	0.35	0.21 (-0.15, 0.57)	0.25	-0.02 (-0.41, 0.37)	0.91

HGU, hepatic glucose uptake; EGP, endogenous glucose production; BMI, body mass index; f, fasting; HOMA-IR, homeostatic model assessment for insulin resistance; M-value, whole-body insulin sensitivity; HbA_1c_, hemoglobin A_1c_; HDL-C,high-density lipoprotein cholesterol; LDL-C, low-density lipoprotein cholesterol; ALT, alanine aminotransferase; AST, aspartate aminotransferase; GGT, γ-glutamyltransferase; MRS, magnetic resonance spectroscopy; LFC, liver fat content; MRI, magnetic resonance imaging.

a = log10 transformed variables.

Model 1 adjusted for age and sex.

Model 2 adjusted for age, sex, and body fat-%.

Bold values represent significance level < 0.05.

## 4 Discussion

In the present study, HGU was not associated with accelerometer-measured habitual SB, PA, or cardiorespiratory fitness. However, we found that EGP was negatively associated with daily standing time, even independently of body adiposity. Additionally, we found that HGU and EGP were associated with body adiposity and whole-body insulin resistance markers (fasting insulin, HOMA-IR, and M-value). We also observed that HGU was negatively associated with daily energy intake of carbohydrates and sugar and positively with daily dietary fiber intake, especially WIDF and unsaturated fatty acids (MUFA and PUFA). Taken together, our results indicate that increasing daily standing time and dietary fiber intake and replacing some of the daily carbohydrates and sugars with unsaturated fat sources might potentially improve hepatic insulin sensitivity. Additionally, we found a beneficial association between EGP with plasma LDL-C, suggesting LDL-C might decrease EGP and thus improve hepatic insulin sensitivity and potentially and paradoxically reduce the risk of type 2 diabetes. These results provide novel insights into the associations between hepatic insulin resistance markers and accelerometer-measured SB and PA, fitness, diet, and metabolic markers in adults with MetS.

### 4.1 Associations of hepatic insulin sensitivity with SB, PA, and fitness

HGU was not associated with SB or any of the PA measures. To the best of our knowledge, no previous studies have investigated the associations between HGU and accelerometer-measured habitual SB and PA. However, previous intervention studies using PET have shown that the intensity and duration of exercise have different effects on HGU. In a recent study, moderate-intensity training was shown to be more effective in improving HGU compared to sprint interval training in adults with normoglycemia or prediabetes/type 2 diabetes (38). However, in another study resistance training did not influence HGU in elderly females (39). Thus, it seems that moderate-intensity aerobic exercise may be the most effective in improving HGU. It is possible, that in the present study, the overall duration and/or intensity of PA were not sufficient to show any associations between HGU and PA. The small variation in PA levels in this homogenous, sedentary, and inactive population may also have prevented detecting associations.

When adjusted for age and sex, EGP was positively associated with SB time in the present study. However, when body fat-% was added to the model the association turned non-significant. Thus, it seems that SB is not independently associated with EGP, and body adiposity might play a more important role in regulating EGP. Although we did not find any associations between HGU and SB or PA, we found that EGP was beneficially associated with daily standing time, independently of body adiposity. To our knowledge, this is a novel finding. However, previous intervention studies have shown that resistance training (39), and treadmill walking (40) have beneficial effects on EGP. In the former study, elderly females performed medium-intensity resistance training three times per week for 4 months, and EGP was suppressed by 28% in the whole group when compared to the baseline results (39). In the latter study, adults with type 2 diabetes walked on a treadmill with medium intensity for 15 weeks (4–5 days/week) and their EGP was suppressed, even if splanchnic glucose uptake was reduced (40). Additionally, in another study, six weeks of MVPA for 20 minutes at least three times per week (60 to 85% of maximal aerobic capacity) resulted in increased insulin sensitivity assessed by both EGP and peripheral glucose uptake in sedentary men (41). Thus, EGP seems to improve with different exercise intensities, such as walking, moderate and vigorous PA, and resistance training. Additionally, we showed in our previous study that standing was favorably associated with whole-body insulin sensitivity (25). Thus, it is plausible to hypothesize that replacing sedentary behavior such as sitting with standing may also have beneficial effects on insulin sensitivity not only at the whole-body level but also at the hepatic tissue level. However, this must be confirmed with intervention studies to show causality.

Aerobic fitness is positively associated with whole-body insulin sensitivity (42, 43), but the role of fitness in hepatic insulin sensitivity is unclear. Our study did not find an association between HGU and cardiorespiratory fitness. When adjusted for sex and age, EGP was negatively associated with fitness. However, when further adjusted for body fat-% all the associations were attenuated. Thus, fitness does not seem to be independently associated with hepatic insulin sensitivity, and body adiposity might play a more important role in regulating liver glucose metabolism.

### 4.2 Associations of hepatic insulin sensitivity with diet

Previous studies have shown that sugar intake, especially fructose (one of the two components of saccharose), has a prominent role in developing NAFLD. Fructose induces fatty liver by two different mechanisms; *de novo* lipogenesis and β-oxidation, which can eventually lead to hepatic insulin resistance (44). In the present study, HGU was inversely associated with the intake of carbohydrates and saccharose of total energy intake (%), which might refer that a diet that consists of lower intakes of carbohydrates, especially sugar, when compared to other macronutrients may improve HGU, and thus hepatic insulin sensitivity in adults with Mets. Interestingly, HGU was not changed after 6 weeks of a very low-calorie diet in adults with obesity (45). However, in the aforementioned study, all meals were replaced with dietary products (carbohydrates 53%, protein 44%, and fat 3%). Thus, potentially different outcomes with different combinations of macronutrients were not assessed.

We also found that HGU was positively associated with the intakes of unsaturated fatty acids MUFA and PUFA. It has been previously shown that MUFA and PUFA may have beneficial effects on glucose and hepatic metabolism with a reduction of HbA1c, glycaemia, and liver fat (46). Additionally, a diet containing high levels of saturated fatty acids had a greater effect on intrahepatic triglyceride content and it increased lipolysis compared to a diet rich in unsaturated fatty acids (47). Although, both of the aforementioned diets increased intrahepatic triglyceride levels, a diet rich in unsaturated fatty acids had more positive effects on liver fat content and lipolysis than a diet with saturated fatty acids (47).

We also detected that HGU was positively associated with dietary fiber intake, especially with WIDF. Previous large prospective studies have shown that high dietary fiber intake, especially insoluble cereal dietary fiber is associated with a reduced risk of type 2 diabetes by potentially improving insulin sensitivity, inflammatory markers, and intestinal microbiota (48). Dietary fiber may also have beneficial effects on metabolic markers related to liver disease and liver cancer, such as high blood glucose, insulin resistance, fatty liver, and MetS (49). Moreover, dietary fiber may have beneficial effects not only on whole-body insulin sensitivity but according to our findings also on hepatic insulin sensitivity. In summary, our results suggest that a decrease in carbohydrates and sugar and an increase in unsaturated fatty acids and daily fiber consumption may have beneficial effects on hepatic insulin sensitivity in adults with MetS. However, this must be confirmed with intervention studies to show causality.

### 4.3 Associations of hepatic insulin sensitivity with cardiometabolic health markers and liver fat content

As mentioned earlier, in the insulin-stimulated state HGU is increased, and EGP is suppressed compared to fasting state in healthy individuals (5). However, in insulin-resistant individuals, the liver fails to increase HGU and suppress EGP adequately in response to insulin stimulation (50). In the present study with adults with MetS, HGU was inversely, and EGP positively associated with body adiposity, fasting insulin, and HOMA-IR. Additionally, HGU was positively, and EGP inversely associated with whole-body insulin sensitivity measured by hyperinsulinemic-euglycemic clamp. Thus, our findings build on the existing evidence and support the notion that obesity and insulin resistance markers are closely associated with hepatic insulin resistance.

Excess fat in the liver affects glucose uptake by taking up space in the hepatocytes, and on the other hand, the fat metabolism intermediates diacylglycerides (DAGs) interfere with insulin signaling (51). Overnutrition and overweight can increase DAG content in the liver due to high delivery of free fatty acids from the circulation or due to increased *de novo* lipogenesis, which both increase intrahepatocellular lipid content, leading to liver insulin resistance (51, 52). A previous study in healthy subjects and type 2 diabetic patients show an inverse association between MRS-measured liver fat content and HGU (53). However, in the present study, we did not find a significant association between HGU, or EGP, and liver fat content measured either with MRS or MRI. It may be so that, fatty acid metabolism intermediates, rather than liver fat content *per se*, may interfere with hepatic glucose metabolism and lead to insulin resistance in the liver.

Finally, we also found that EGP was inversely associated with LDL-C. Even though this beneficial association may not sound expected because high plasma LDL-C levels are closely associated with an increased risk of cardiovascular disease (54), it is in accordance with recent findings regarding LDL-C and glucose metabolism. An increase in LDL-C has been found to be positively associated with insulin secretion (55), and inversely with the risk of type 2 diabetes (56). A meta-analysis found that cholesterol-lowering drugs slightly increased the risk of developing type 2 diabetes (57). Our results suggest that the inverse association of EGP with LDL-C might indicate that LDL-C decreases EGP, thus improving hepatic insulin sensitivity and paradoxically reducing the risk of type 2 diabetes in adults with MetS. To our knowledge, this is a novel result and may be one possible mechanistic explanation for the reduced risk of type 2 diabetes. However, this must be confirmed with intervention studies to show causality.

### 4.4 Strengths and limitations

A major strength of the current study is the use of the gold standard euglycemic-hyperinsulinemic clamp method (22) for measuring insulin sensitivity, combined with PET imaging to measure glucose uptake directly in the liver. PET represents the current gold standard for assessing non-invasive tissue-specific glucose uptake *in vivo* (58). Further advantages are the use of accelerometers and validated algorithms for measuring SB and PA (26, 27) for four consecutive weeks, as well as the measurement of cardiorespiratory fitness with direct respiratory gas measurements. Limitations include a relatively small sample size, but the techniques, especially PET imaging, used in this study prevent from using larger groups. Also, a limitation would be the medications some of the participants used which might have affected the results. The key limitation of the present study is the cross-sectional setting, which prevents the causal interpretation of these results. Therefore, future studies should aim to assess the relationship between HGU, EGP and habitual SB, PA, and other lifestyle factors in longitudinal and experimental settings.

### 4.5 Conclusions

Taken together the results of the present study show that dietary factors may be more important than daily habitual physical (in) activity or aerobic fitness for the healthy liver. The negative association between HGU and daily carbohydrates and saccharose intake, and the positive associations between MUFA, PUFA, and WIDF suggest that replacing some of the daily carbohydrates and sugars with quality fats, as well as adding fiber to the diet might result in a healthier liver. However, interventions investigating the impact of dietary modification on hepatic insulin sensitivity are warranted. On the other hand, SB, PA, or fitness were not associated with HGU, and standing was the only parameter associated with insulin sensitivity markers (EGP). This suggests that increasing standing in daily life could lead to healthier liver glucose metabolism, but this also needs to be confirmed in targeted intervention studies. Our results also confirm unhealthy body composition is associated with impaired hepatic insulin sensitivity markers. Additionally, the beneficial association between EGP and plasma LDL-C, suggests that LDL-C may decrease EGP and thus improve hepatic insulin sensitivity and paradoxically reduce the risk of type 2 diabetes.

## Data availability statement

The original contributions presented in the study are included in the article/supplementary material. Further inquiries can be directed to the corresponding author.

## Ethics statement

The studies involving humans were approved by Ethics Committee of the Hospital District of Southwest Finland. The studies were conducted in accordance with the local legislation and institutional requirements. The participants provided their written informed consent to participate in this study.

## Author contributions

SL: Data curation, Formal Analysis, Funding acquisition, Investigation, Visualization, Writing – original draft, Writing – review & editing. TS: Conceptualization, Data curation, Formal Analysis, Funding acquisition, Investigation, Writing – review & editing. TG: Data curation, Formal Analysis, Investigation, Writing – review & editing. MH: Formal Analysis, Supervision, Writing – review & editing. EL: Formal Analysis, Supervision, Writing – review & editing. OE: Methodology, Writing – review & editing. MS: Investigation, Writing – review & editing. PK: Investigation, Writing – review & editing. MK: Investigation, Writing – review & editing. HV: Data curation, Formal Analysis, Methodology, Writing – review & editing. HS: Formal Analysis, Methodology, Writing – review & editing. TV: Conceptualization, Writing – review & editing. JH: Investigation, Writing – review & editing. KL: Conceptualization, Writing – review & editing. NH: Data curation, Investigation, Writing – review & editing. KK: Conceptualization, Writing – review & editing. VS: Conceptualization, Formal Analysis, Investigation, Writing – review & editing. JK: Conceptualization, Writing – review & editing. IH: Conceptualization, Formal Analysis, Funding acquisition, Investigation, Project administration, Resources, Supervision, Writing – review & editing.
